# Effects of Separate Cognitive Training on Endurance Exercise Performance

**DOI:** 10.3390/jfmk10040391

**Published:** 2025-10-08

**Authors:** Neil Dallaway, Steven R. Bray, Kira L. Innes, Kathryn E. Andrusko, Christopher Ring

**Affiliations:** 1 School of Sport, Exercise & Rehabilitation Sciences, University of Birmingham, Birmingham B15 2TT, UK; 2 Department of Kinesiology, McMaster University, Hamilton, ON L8S 4L8, Canada

**Keywords:** cognitive training, endurance exercise, training

## Abstract

**Background:** Combined cognitive and physical training develops resilience to mental fatigue, reduces perceived effort, and improves endurance exercise performance when compared to physical training and no training. The isolated contribution of cognitive training toward endurance performance has yet to be determined. Accordingly, we examined the effects of separate cognitive training on endurance exercise performance. **Method:** Two studies employed a pre-test/training/post-test design, with participants randomly assigned to cognitive training or control groups. At pre-test and post-test, participants completed a rhythmic handgrip task (Study 1) or a graded exercise test on a cycle ergometer (Study 2). In Study 1, the cognitive training group completed 20 sessions (four 20 min sessions per week for five weeks) of cognitive training (incongruent Stroop and 2-back tasks), whereas the control group completed no training. In Study 2, the cognitive training group completed nine sessions (three 10 min sessions per week for three weeks) of cognitive training (incongruent Stroop, stop-signal and typing inhibition tasks), whereas the control group completed nine sessions of sham training (congruent Stroop, sham stop-signal and sham typing inhibition tasks). Endurance exercise performance was measured as force production (Study 1) and time to exhaustion (Study 2). Heart rate, exertion and fatigue were also measured. **Results:** Endurance performance, indexed by force production (Study 1) and time to exhaustion (Study 2), did not change from pre-test to post-test and did not differ between cognitive training and control groups. Similarly, ratings of perceived exertion and heart rate during the exercise tasks did not differ between cognitive training and control groups (Studies 1 and 2). **Conclusions:** Since separate cognitive training did not improve exercise endurance performance, combined training should be used to create a synergistic training stimulus for brain adaptation and performance enhancement.

## 1. Introduction

Endurance, defined as the ability to sustain prolonged effort and resist fatigue, is important for athletes wishing to perform optimally in competitive sport. Accordingly, athletes and coaches employ training methods to increase effort and fatigue resilience and thereby enhance endurance performance. Mental fatigue, a low arousal negative valence psychobiological state induced by prolonged cognitive effort, has emerged as an important negative factor that can impair sport and exercise performance [1,2,3,4]. Based on the psychobiological model of exercise endurance, Marcora and colleagues [5] attribute this fatigue-related impairment in performance to a central mechanism (i.e., perceived effort) rather than peripheral mechanism (i.e., physiological homeostatic failure) favored by others [6,7]. Marcora and colleagues developed *Brain Endurance Training (BET)*—a training method that combines cognitive and exercise tasks—to enable athletes to recalibrate perceived effort, develop resilience to mental fatigue, and improve endurance exercise performance [8]. Research studies confirm that BET improves sport and exercise performance [8,9,10,11,12,13,14,15]. Importantly, evidence shows that BET-related performance improvements are accompanied by changes in perception of effort (e.g., [15]), whereby a standard exercise intensity is perceived as being easier to perform after completing weeks of BET.

Nonetheless, an important gap in our understanding of the effects of combined cognitive and physical training on endurance performance and associated psychobiological processes is the relative contribution of cognitive training and physical training to the training-related benefits, especially for performance. In other words, can athletes separately train their cognitive and physical abilities or do they need to train them together to create a greater stimulus to encourage neuroplasticity and thereby achieve synergistic benefits. This is important because, if proven effective, separate cognitive training could be used by athletes to fill gaps in their training programs that supplement or substitute their standard physical training regimes. This may be especially important when they are unable to physically train, which may happen because of injury, travel, and resource unavailability. To date, no published study has reported on the effects of separate cognitive training on endurance exercise performance in young athletes. The evidence provided by research in older adults and patient groups comparing the effects of separate and combined cognitive and physical training is mixed and inconclusive [16,17]. Accordingly, empirical research is needed to determine whether cognitive training alone can improve endurance exercise performance.

Training one task can improve the ability to perform another task. Studies have provided evidence to support this general transfer of training, perhaps via adaptation to mental workload. One such study examined the effect of physical training on mental and physical performance [18]. At pre-test and post-test, participants performed a cognitive task (Stroop) and a subsequent exercise task (graded cycle ergometer test). The two-week training comprised a maximal endurance handgrip squeeze twice a day (intervention) or no activities (control). Exercise endurance, measured by cycling time to exhaustion, improved following handgrip training but not in the control group. Another study examined the effect of exercise training on time trial performance following a battery of cognitive tasks. The training comprised cycling (three 60 min submaximal sessions per week for five weeks) or no activity (control). Exercise endurance, measured by distance covered, improved following training but not in the control group, thereby suggesting that physical training improved tolerance to mental load [19]. These studies suggest that training in one domain may increase the ability (actual or perceived) to cope with psychobiological demands.

The current research project examined the effects of cognitive training on handgrip (Study 1) and cycling (Study 2) endurance performance. Our study purposes were twofold. First, we investigated whether separate cognitive training improves endurance when compared to the control group. We hypothesized that cognitive training would improve endurance exercise performance when compared to the control group. Second, we explored changes in psychobiological responses as a function of training. We hypothesized that cognitive training would reduce fatigue and exertion (i.e., psychological factors) but would not change heart rate (i.e., physiological factor).

## 2. Method

### 2.1. Study 1

#### 2.1.1. Participants

Participants were 22 (15 females, 7 males) healthy undergraduate students aged 22.0 ± 2.0 years who received a GBP 20 voucher and course credit. They were randomly assigned to one of two groups: cognitive training (*n* = 14) or control (*n* = 8). Participants were asked to have a regular night’s sleep (>7 h) and abstain from exercise and alcohol consumption (24 h), caffeine (3 h), and food (1 h) before each session. Exclusion criteria included dominant hand injury, upper body strength training, and changes in habitual exercise during the study. The protocol was approved by our university ethics committee (University of Birmingham ethics committee, SPP 17102019-2 approved 7 November 2017). Participants gave written informed consent. Power calculations using GPower [20] indicated that with a sample size of 22 the study was powered at 80% to detect significant (*p* < 0.05) interaction effects (f = 0.31, η^2^ = 0.09) corresponding to a small-to-medium effect size by analysis of variance [21].

#### 2.1.2. Design

The study adopted a pre-test/training/post-test design, with one between-participant factor (group: cognitive training, control) and two within-participant factors (test: pre-test, post-test; task: before 2-back, after 2-back). Cognitive training participants attended 22 sessions over seven weeks, comprising a pre-test (week 1), 20 training sessions (weeks 2–6), and a post-test (week 7). Control participants only attended the pre-test session at week 1 and post-test session at week 7.

#### 2.1.3. Maximum Voluntary Contraction

Participants squeezed a handgrip dynamometer as hard as possible [22]. The dynamometer [23] was held in their dominant hand with their arm flexed at 100 degrees. Each maximal contraction was followed by a 1 min rest and repeated three times. The highest peak force was their maximum voluntary contraction (MVC).

#### 2.1.4. Exercise Task

Participants completed a 5 min rhythmic handgrip task. They squeezed the dynamometer with their dominant hand once a second (1 Hz) cued by an audio metronome. They completed a 1 min familiarization task with visual performance feedback. A standardized script was read to participants before the task, at 150 s, and at 270 s, instructing them to “generate as much force as possible in the timeframe for a chance of winning a GBP 20 voucher”. Signals were measured via a Power 1401 (CED, Cambridge, UK) analog-to-digital converter (16-bit, sampling 2.5 kHz) and the output recorded using Spike-2 software (v6.06). Performance was determined by the average force in Newtons per second (N/s).

#### 2.1.5. Cognitive Tasks

The 2-back task [24] involves sustained attention and memory updating [25]. In this 20 min task, participants were shown a continuous series of random consonants and indicated if the current letter displayed was the same as (target) or different from (non-target) the one presented two letters earlier. The letters were displayed, once every 3 s for 500 ms. Participants used their non-dominant hand to press the number “1” key on a keyboard if the current letter displayed was the same as the letter two prior, and the number “2” key if it was different. The targets to non-target ratio was 2:1. Percentage of correct responses assessed task performance.

The incongruent color word Stroop task involves sustained attention and response inhibition. In this 20 min task, participants were required to indicate the font color (red, yellow, green, blue) of a color word from two possible answers displayed in a black font at the bottom left and right corners of the display with either a left or right mouse click. Participants received verbal and written instructions prior to the training task. The Stroop test requires response inhibition and working memory. For all Stroop tasks, the stimulus was presented for 2500 ms or until a response was given, followed by a fixation cross for 500 ms. The sequence and increasing difficulty of the cognitive tasks were designed to mitigate any learning effects.

The incongruent number Stroop task involves sustained attention and response inhibition. In this novel 5 min task, participants were required to indicate how many words were displayed from a list of number words (one, two, three, four) of the same type (e.g., two, two, two); the correct answer was the number of words in the list (e.g., three) whereas the incorrect answer was the number that the words represented (e.g., two). The two possible answers (e.g., two or three) were displayed in black font at the bottom left and right corners of the display, and participants responded with either a left (Z) or right (/) keyboard button press. They received verbal and written instructions prior to the task. Performance was measured by speed (reaction time, ms) and accuracy (% correct).

#### 2.1.6. Training

The cognitive training group completed 20 cognitive training sessions (4 sessions per week for 5 weeks) consisting of the 2-back task and color word incongruent Stroop task. In each session, cognitive training participants completed a 20 min 2-back task or incongruent Stroop task. They completed a 2-back task on sessions 1–4 (week 1), 9–12 (week 3), and 17–20 (week 5). To create progressive cognitive overload, the 2-back task’s letter refresh rate increased every four sessions across training: 3000 ms (sessions 1–4), 2500 ms (sessions 9–12), and 2000 ms (sessions 17–20). They completed an incongruent color word Stroop task on sessions 5–8 (week 2) and 13–16 (week 4). No task performance feedback was provided. The control group did not complete any training activities and acted as a passive control condition. All cognitive tasks, in both the testing and training sessions, were implemented using E-Studio 2.0 (Psychology Software Tools, Pittsburg, PA, USA).

#### 2.1.7. Measures

Ratings of perceived exertion (RPE) were obtained using an 11-point CR-10 scale [26], anchored with the descriptors “nothing at all” and “maximal”. The standard instructions for the scale [27] were read out to participants. Mental fatigue and mental exertion were rated on 11-point category ratio (CR-10) scales. The mental fatigue scale was anchored with the extreme descriptors “nothing at all” and “totally exhausted”. The mental exertion scale was anchored with the extreme descriptors “nothing at all” and “maximal mental exertion”. An electrocardiogram was recorded using surface electrodes in a chest configuration and amplifier (509, Morgan, Petersfield, UK). The R-wave was scored and used to compute RR intervals and heart rate (beats per minute).

#### 2.1.8. Procedure

In the pre-test and post-test sessions, participants completed the 5 min exercise task twice: before (first exercise task) and after (second exercise task) completing a 20 min 2-back cognitive task. In the post-test session, they also completed a 5 min novel incongruent number Stroop cognitive task after the second exercise task. This novel cognitive task was included in the post-test session in order to determine whether cognitive training with one battery of tasks improves performance on a different cognitive task (i.e., to assess the generalizability of training). Participants provided a rating of perceived exertion every 60 s of the exercise task, ratings of mental fatigue at baseline and immediately after each task, and ratings of mental exertion immediately after each task. In the five weeks between testing sessions, they completed cognitive tasks (cognitive training group) or rested (control group).

#### 2.1.9. Statistical Analysis

Mixed factorial ANOVAs were performed using SPSS 30 software on the measures collected during or after the exercise and cognitive tasks. Partial eta-squared (η_p_^2^) was reported as a measure of effect size [21]. Significance was set at *p* < 0.05.

### 2.2. Study 2

#### 2.2.1. Participants

Participants were 22 (12 females, 10 males) healthy undergraduate students aged 18.8 ± 1.3 years who received a voucher for 20 Canadian dollars and course credit. The sample pool was stratified by sex and randomly assigned to one of two groups: cognitive training (*n* = 11) or control (*n* = 11). Participants were asked to eat similar meals and abstain from caffeine and sport supplements prior testing and training sessions. They were also asked to maintain their habitual exercise during the study. The protocol was approved by our university ethics committee (McMaster University ethics committee, MREB 2015-159 approved 1 December 2015). Participants gave written informed consent. GPower 3.1 software [20] indicated that the study was powered at 80% to detect significant interaction effects with small-to-medium effect sizes (*f* = 0.31, *η*^2^ = 0.09) by analysis of variance [21].

#### 2.2.2. Design

The study adopted a pre-test/training/post-test design, with one between-participant factor (group: cognitive training, control) and one within-participant factor (test: pre-test, post-test). Participants attended 12 sessions over 29 days, comprising a familiarization (day 1), pre-test (day 8), nine training sessions (days 9–28), and a post-test (day 29).

#### 2.2.3. Exercise Task

Participants completed a graded exercise test on a cycle ergometer (Lode, Corival 5.5, Groningen, The Netherlands). The resistance on the ergometer followed an upward ramp that was independent of cadence. The test began with a 2 min warm-up with 50 W resistance that was followed by a progressive increase in workload (+1 W every 3 s). Participants were provided continuous feedback about their cadence (revolutions per minute, rpm) throughout the task and were instructed to maintain cadence between 70 and 80 rpm and to continue pedaling for as long as possible. The test terminated when: cadence fell below 60 rpm for more than 5 s, cadence was less than 70 rpm for 30 s, or the participant stopped pedaling. When cadence dropped below 70 rpm, participants were reminded by the experimenter to “keep the rpm above 70”. No other instruction was given to participants. Participants were unaware of the time elapsed and the total workload throughout the protocol. Time to exhaustion in seconds assessed performance.

#### 2.2.4. Cognitive Tasks

The 10 min incongruent Stroop task required participants to say aloud the color of the font in which the word was presented, while ignoring the semantic meaning of the word (e.g., for the word “blue” displayed in green font, the participant would respond “green”). In all trials the font color mis-matched the text (e.g., the word “blue” was presented in green font). Presentation software (Version 18.1) was used to have the words appear one at a time on a 15” screen, with each word being displayed for 1200 ms in the center of the screen. This task draws on inhibitory control because participants were required to ignore the word and inhibit the instinctive reaction to read aloud the word to respond correctly.

The 10 min stop-signal task comprised four blocks: one 10-trial practice block and three 30-trial experimental blocks. The task was presented on a 17” monitor. Each trial began with participants fixating on a white “+” in the middle of a black screen followed by either a white square or white circle. Participants were instructed to respond as quickly and as accurately as possible to whichever of the two stimuli appeared by pressing a button on the keyboard that corresponded with either of the answers, “z” and “/”, respectively. During 75% of the trials, the stimuli appeared in silence; in the other 25% of the trials, a stop-signal delay was presented as an auditory stimulus (beep). These stop-signal delay trials occurred randomly throughout the trials. Participants were instructed to inhibit their responses by not pressing either of the keys during stop-signal delay trials.

The 10 min typing inhibition task provided participants with a passage from a novel, which they were asked to transcribe while omitting the two most common characters in the English language: “space” and “e”. Typing is a highly automatic task that, when altering habitual performance, requires response inhibition. Participants were instructed not to fix any mistakes. Additionally, participants transcribed the passage in white font to prevent visual feedback of what was being typed. The participants were instructed to transcribe as much of the passage as they could, and make as few errors as possible.

The 10 min congruent Stroop task presented words with matched font colors (e.g., the word “blue” was presented in blue font) on a 15” screen, with each word displayed for 1200 ms. Participants were instructed to say aloud the color of the font in which the words were presented, while ignoring the semantic meaning of the word; however, in this case the semantic meaning and the font color were matched (e.g., for the word “blue” displayed in blue font, the participant would respond “blue”). The number of errors made were recorded.

The 10 min sham stop-signal task, unlike the stop-signal task (see above), had no additional auditory stimulus and thus no “stop” requiring response inhibition. Participants were required to respond as quickly and as accurately as possible by pressing one of two keys on a keyboard depending on which stimulus was presented. Participants pressed “z” if they saw a white square and “/” if they saw a white circle. This version of the task does not require response inhibition as participants are not required to override the learned reaction.

The 10 min sham typing inhibition task provided participants with the same passage as the typing inhibition task (see above) and they transcribed the text verbatim. Participants were instructed not to fix any mistakes. Additionally, participants transcribed the passage in white font to prevent visual feedback of what was being typed. The participants were instructed to transcribe as much of the passage as they could, and make as few errors as possible. This version of the typing inhibition task was thought to not require inhibition control because participants are not preventing themselves from typing in a habitual manner.

#### 2.2.5. Training

Both groups completed nine cognitive training sessions over three weeks (i.e., three sessions per week). In each session, they completed one of three 10 min tasks. Each task was completed during three separate training sessions. Cognitive training participants completed one of three 10 min cognitive tasks that required response inhibition: incongruent Stroop task, stop-signal task, or typing inhibition task (see above). Control participants completed one of three 10 min cognitive tasks that did not require response inhibition: congruent Stroop task, sham stop-signal task, or sham typing inhibition task (see above). No task performance feedback was provided.

#### 2.2.6. Measures

Ratings of perceived exertion (RPE) were obtained using a 15-point scale [26], anchored with the descriptors “6 = no exertion at all” and “20 = maximal exertion”. Mental exertion was rated using an 11-point category ratio (CR-10) scale, anchored with the descriptors “0 = nothing at all” and “10 = maximal mental exertion”. The standard instructions for the scale [27] were read to participants prior to the task. Heart rate (beats per minute) was measured using a chest sensor and watch (Polar Electro N2965 and S625X, Kempele, Finland).

#### 2.2.7. Procedure

In the pre-test and post-test sessions, participants completed the graded exercise test. Each participant provided a rating of perceived exertion, and their heart rate was recorded every 60 s of the exercise task. In each training session during the 3-week training intervention, they completed a 10 min cognitive task and provided ratings of mental exertion after each cognitive task (see above).

## 3. Results

### 3.1. Study 1

#### 3.1.1. Testing

**Figure 1:**
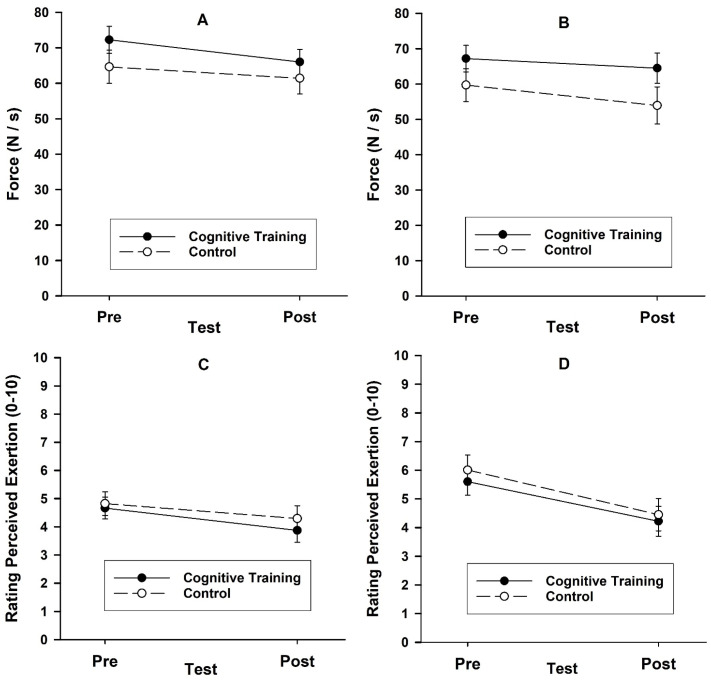
Mean (*SE*) force production during the first exercise task (**A**) and second exercise task (**B**) and the rating of perceived exertion for the first exercise task (**C**) and second exercise task (**D**) as a function of group (cognitive training, control) and test (pre, post) in Study 1.

A 2-group (cognitive training, control) by 2-sex (male, female) by 2-test (pre, post) by 2-task (first exercise task, second exercise task) ANOVA on force production during the exercise task (Figure 1A,B) did not reveal any group effects; notably, no group by test interaction, *F*(1, 18) = 0.00, *p* = 1.00, η_p_^2^ = 0.00, and no group by test by task interaction, *F*(1, 18) = 2.95, *p* = 0.10, η_p_^2^ = 0.14. The test effect was not significant, *F*(1, 18) = 2.67, *p* = 0.12, η_p_^2^ = 0.13; participants produced similar force during the first (*M* = 65.97 N/s) and second (*M* = 61.48 N/s) tests. The only significant effect was for task, *F*(1, 18) = 17.94, *p* < 0.001, η_p_^2^ = 0.50; participants produced less force during the second exercise task (*M* = 61.36 N/s) than the first exercise task (*M* = 66.09 N/s). A 2-group by 2-sex by 2-test ANOVA on the maximum voluntary contraction did not reveal any effect for group, *F*(1, 18) = 0.20, *p* = 0.66, η_p_^2^ = 0.01, (*M*_cognitive training_ = 377 N, *M*_control_ = 372 N), test, *F*(1, 18) = 0.02, *p* = 0.89, η_p_^2^ = 0.00, (*M*_pre-test_ = 374 N, *M*_post-test_ = 375 N), or group by test, *F*(1, 18) = 0.14, *p* = 0.72, η_p_^2^ = 0.01.

A 2-group by 2-sex by 2-test by 2-task (first exercise task, second exercise task) ANOVA on the ratings of perceived exertion (Figure 1C,D) yielded no effects for group, *F*(1, 18) = 1.37, *p* = 0.26, η_p_^2^ = 0.07, group by test, *F*(1, 18) = 1.87, *p* = 0.19, η_p_^2^ = 0.09, or group by test by task, *F*(1, 18) = 0.91, *p* = 0.35, η_p_^2^ = 0.05. There was only a main effect for test, *F*(1, 18) = 13.01, *p* = 0.002, η_p_^2^ = 0.42, with perceived exertion lower in the post-test (*M*_post-test_ = 4.21) than the pre-test (*M*_pre-test_ = 5.28).

A 2-group by 2-sex by 2-test by 2-task (first exercise task, second exercise task) ANOVA on heart rate yielded no main effects for group, *F*(1, 13) = 3.43 *p* = 0.10, η_p_^2^ = 0.23, (*M_cognitive training_* = 78 bpm, *M*_control_ = 84 bpm), test, *F*(1, 13) = 1.56, *p* = 0.24, η_p_^2^ = 0.12, (*M*_pre-test_ = 83 bpm, *M*_post-test_ = 80 bpm), and task, *F*(1, 13) = 1.51, *p* = 0.25, η_p_^2^ = 0.12, (*M*_first exercise task_ = 82 bpm, *M*_second exercise task_ = 80 bpm), as well as no interaction effects involving group.

Mental fatigue was examined using a 2- group by 2-sex by 2-test by 4-task (baseline, first exercise task, 2-back task, second exercise task) ANOVA. The analysis yielded no effects for group, *F*(1, 18) = 0.02, *p* = 0.89, η_p_^2^ = 0.00, test, *F*(1, 18) = 0.00, *p* = 98, η_p_^2^ = 0.00, group by test, *F*(1, 18) = 0.25, *p* = 0.63, η_p_^2^ = 0.01, or group by test by task, *F*(3, 16) = 0.03, *p* = 0.86, η_p_^2^ = 0.00. There was a main effect for task, *F*(3, 16) = 33.12, *p* < 0.001, η_p_^2^ = 0.86, whereby mental fatigue at baseline (*M*_baseline_ = 2.04) and after the first exercise task (*M*_first exercise task_ = 2.09) were lower than after the 2-back task (*M*_2-back task_ = 4.80) and after the second exercise task (*M*_second exercise task_ = 4.11).

Mental exertion was examined using a 2-group by 2-sex by 2-test by 3-task (first exercise task, 2-back task, second exercise task) ANOVA. The analysis yielded no effects for group, *F*(1, 18) = 0.01, *p* = 0.94, η_p_^2^ = 0.00, test, *F*(1, 18) = 0.66, *p* = 43, η_p_^2^ = 0.04, group by test, *F*(1, 18) = 3.48, *p* = 0.08, η_p_^2^ = 0.16, or group by test by task, *F*(2, 17) = 0.60, *p* = 0.56, η_p_^2^ = 0.07. There was a main effect for task, *F*(2, 17) = 36.19, *p* < 0.001, η_p_^2^ = 0.81; mental exertion was greater for the 2-back task (*M*_2-back_ = 5.07) than the first (*M*_first exercise task_ = 2.37) and second (*M*_second exercise task_ = 3.20) exercise tasks. Perceived exertion was higher during the second exercise task compared to the first exercise task.

#### 3.1.2. Training

Performance on the 2-back task, indexed by the percent correct responses, was examined using a 2-group by 2-sex by 2-test ANOVA. This analysis did not reveal any effect for group, *F*(1, 18) = 1.03, *p* = 0.33, η_p_^2^ = 07, (*M_cognitive training_* = 94%, *M*_control_ = 91%), test, *F*(1, 18) = 3.07, *p* = 0.10, η_p_^2^ = 0.19, (*M*_pre-test_ = 91%, *M*_post-test_ = 94%), or group by test, *F*(1, 18) = 1.15, *p* = 0.30, η_p_^2^ = 0.08. Performance on the number Stroop task (post-test session only) was examined using 2-group by 2-sex ANOVAs. These analyses yielded no group effects for percent correct performance, *F*(1, 18) = 0.02, *p* = 0.89, η_p_^2^ = 0.00, (*M*_cognitive training_ = 88%, *M*_control_ = 88%), or reaction time, *F*(1, 18) = 1.45, *p* = 0.25, η_p_^2^ = 0.07, (*M*_cognitive training_ = 786 ms, *M*_control_ = 921 ms).

### 3.2. Study 2

#### 3.2.1. Testing

**Figure 2:**
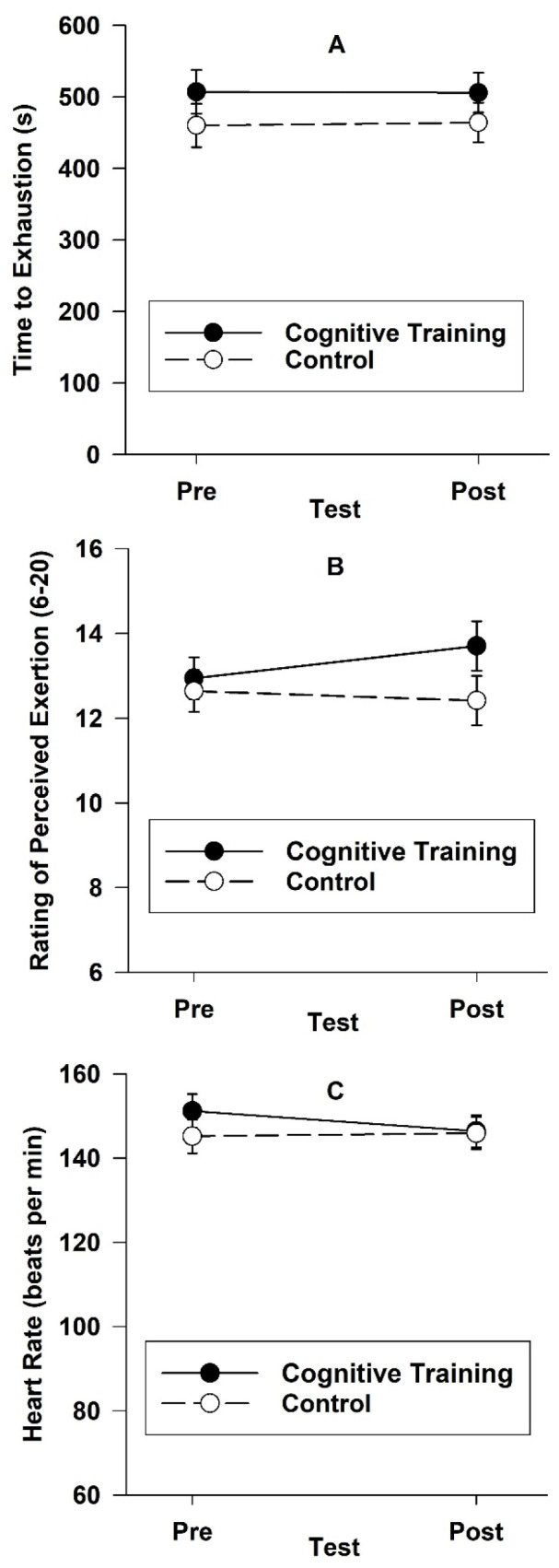
Mean (*SE*) time to exhaustion (**A**), rating of perceived exertion (**B**), and heart rate (**C**) for the exercise task as a function of group (cognitive training, control) and test (pre, post) in Study 2.

A 2-group (cognitive training, control) by 2-sex (male, female) by 2-test (pre, post) ANOVA on time to exhaustion on the exercise task (Figure 2A) revealed no effects for group, *F*(1, 18) = 1.18, *p* = 0.29, η_p_^2^ = 0.06, test, *F*(1, 18) = 0.14, *p* = 0.72, η_p_^2^ = 0.01, or group by test, *F*(1, 18) = 0.43, *p* = 0.52, η_p_^2^ = 0.02. A 2-group by 2-sex by 2-test ANOVA on ratings of perceived exertion (Figure 2B) yielded no effects for group, *F*(1, 18) = 1.36, *p* = 0.26, η_p_^2^ = 0.07, test, *F*(1, 18) = 0.61, *p* = 0.44, η_p_^2^ = 0.03, and group by test, *F*(1, 18) = 2.08, *p* = 0.17, η_p_^2^ = 0.10. A 2-group by 2-sex by 2-test ANOVA on average heart rate (Figure 2C) yielded no effects for group, *F*(1, 18) = 0.40, *p* = 0.54, η_p_^2^ = 0.02, test, *F*(1, 18) = 0.96, *p* = 0.34, η_p_^2^ = 0.05, and group by test, *F*(1, 18) = 1.79, *p* = 0.20, η_p_^2^ = 0.09.

#### 3.2.2. Training

A series of 2-group by 2-sex ANOVA were conducted on average (across three sessions) performance during each of the three cognitive tasks. They yielded group effects for errors on the Stroop task, *F*(1, 18) = 57.00, *p* < 0.001, η_p_^2^ = 0.76, (*M*_cognitive training_ = 44.20 ± 19.16, *M*_control_ = 0.48 ± 0.86), reaction times on the stop-signal task, *F*(1, 18) = 21.62, *p* < 0.001, η_p_^2^ = 0.55, (*M*_cognitive training_ = 595 ± 121 ms, *M*_control_ = 416 ± 40 ms), and number of characters typed on the typing inhibition task, *F*(1, 18) = 11.27, *p* < 0.003, η_p_^2^ = 0.39, (*M*_cognitive training_ = 1188 ± 163, *M*_control_ = 1864 ± 648). Moreover, a 2-group by 2-sex ANOVA yielded a group main effect for group for averaged (across nine sessions) ratings of mental exertion, *F*(1, 18) = 17.02, *p* < 0.003, η_p_^2^ = 0.49, (*M*_cognitive training_ = 5.23 ± 1.35, *M*_control_ = 2.74 ± 1.47).

## 4. Discussion

We investigated the effects of separate cognitive training on subsequent endurance exercise performance. Contrary to expectation, cognitive training alone did not improve exercise endurance when compared to the control groups for both handgrip (Study 1) and cycling (Study 2) tasks. Specifically, Study 1 found that force production when performing a rhythmic handgrip task fell by 5% following five weeks of cognitive training compared to a fall of 10% following no training control. Study 2 found that time to exhaustion while performing a graded exercise ergometer test lasted 1% longer following three weeks of cognitive training, compared to a 4% decrease following sham control training. Taken together, these findings suggest that cognitive training needs to be combined with exercise training to subsequently improve exercise endurance performance [8,16,17].

It is worth noting that cognitive training changed perception and/or performance during the cognitive tasks. In Study 1, five weeks of cognitive training (compared to no training) was associated in the post-test session with decreased mental fatigue and exertion coupled with faster responses during the cognitive tasks, which notably imposed response inhibition (Stroop) or memory updating (2-back) executive function operations, compared to the no training control. Thus, training increased resilience and improved cognition. In Study 2, three weeks of cognitive training was associated with more mental fatigue in the training sessions, coupled with worse speed and accuracy during the cognitive tasks (Stroop, stop-signal, typing), all of which included response inhibition demands, compared to the control group. Thus, compared to sham training, active cognitive training was more cognitively demanding and more mentally fatiguing. These findings indicate that the cognitive task training interventions achieved their intended effects (i.e., to induce and build resilience to mental fatigue by repeatedly inducing a state of mental fatigue). It is also worth noting that, in line with past research, Study 1 also confirmed that performing a 20 min 2-back task increased mental fatigue, and moreover, impaired endurance performance on a subsequent exercise task during testing [10,11]. In other words, mental fatigue acutely impaired subsequent exercise, in accordance with the broader research literature on the fatigue–performance relationship [1].

### 4.1. Study Limitations and Future Directions

The null study findings should be interpreted after considering potential methodological limitations. First, the sample sizes were relatively small. It is worth noting that they were broadly comparable with most previous combined training studies [8,9,10,11,12,13,14,15]. Moreover, the effect sizes for the group by test interaction effects for our measures of exercise endurance performance were none (Study 1, η_p_^2^ = 0.00) and small (Study 2, η_p_^2^ = 0.02), which argues against there being effects that merely required a few more participants to be detected. Second, training lasted five weeks (Study 1) and three weeks (Study 2) and comprised twenty 20 min sessions (Study 1) and nine 10 min sessions (Study 2). Accordingly, training over extended periods and/or for longer sessions may be necessary to uncover a beneficial effect of cognitive training on exercise performance. Research with larger samples and higher doses of cognitive training could assess the effects of cognitive training on physical performance. However, our findings argue that cognitive training *on its own* is not an effective training stimulus for developing endurance during exercise [16,17]. Evidence that combined cognitive and exercise training to improve endurance exercise performance [8,9,10,11,12,13,14,15] suggests that the mechanisms underlying the effects of BET on physical performance are related to central synergistic neuroplastic adaptations brought about by repeated exposure to “higher-than-normal” training stimuli associated with combining “normal” physical and cognitive training loads [10,11]. This interpretation aligns with contemporary principles of athletic training [28] as well as evidence that maximal physical performance is limited by central rather than peripheral mechanisms [29,30], see also [31].

### 4.2. Practical Applications

Despite its questionable utility as a separate form of training to improve sport and exercise performance, cognitive training may serve other purposes, such as providing a safe training stimulus for athletes and exercisers who are unable to train physically due to injury or illness or situation. Given that cognitive training increases mental exertion and mental fatigue, repeated exposure to cognitive training might help maintain endurance exercise performance by preserving the status of the central nervous system that mediates experiences of perceived exertion that might otherwise deteriorate when physical training that normally stimulates those areas is not possible.

### 4.3. Conclusions

The current studies, which examined the effects on performance from repeatedly completing mentally fatiguing cognitive tasks without being coupled with physical training, showed that separate cognitive training did not improve subsequent exercise endurance. Accordingly, athletes should employ combined cognitive plus physical training protocols to create a sufficiently large synergistic training stimulus for brain adaptation and performance enhancement.

## Data Availability

The data are available from the corresponding author on request. They are available in the UBIRA eData repository (https://edata.bham.ac.uk, accessed on 24 June 2025).
